# Mixed invasive ductal and lobular carcinoma has distinct clinical features and predicts worse prognosis when stratified by estrogen receptor status

**DOI:** 10.1038/s41598-017-10789-x

**Published:** 2017-09-04

**Authors:** Yi Xiao, Ding Ma, Miao Ruan, Shen Zhao, Xi-Yu Liu, Yi-Zhou Jiang, Zhi-Ming Shao

**Affiliations:** 1 Department of Breast Surgery, Fudan University Shanghai Cancer Center; Cancer Institute, Fudan University Shanghai Cancer Center, 270 Dong-an Road, Shanghai, 200032 People’s Republic of China; 2 Department of Oncology, Shanghai Medical College, Fudan University, Shanghai, People’s Republic of China; 3 Department of Pathology, Fudan University Shanghai Cancer Center, Shanghai, People’s Republic of China; 4 Institutes of Biomedical Sciences, Fudan University, Shanghai, People’s Republic of China

## Abstract

In order to investigate clinicopathological characteristics and prognosis of mixed invasive ductal and lobular carcinoma (IDC-L), 209,109 primary breast cancer patients diagnosed with invasive ductal carcinoma (IDC), invasive lobular carcinoma (ILC) or IDC-L were included. It was found that IDC-L patients had lower tumor grade and higher hormone receptor positive proportions than IDC patients. Moreover, IDC-L patients were younger and had a similar hormone receptor status compared with ILC patients. Kaplan-Meier plots showed that the breast cancer-specific survival (BCSS) of IDC-L patients was significantly better than IDC patients (P < 0.001) and tended to be better than ILC patients (P = 0.166). However, after adjusting for clinicopathological factors, survival advantage of IDC-L disappeared. Subgroup analysis indicated that IDC-L had higher hazard ratios (HRs) than IDC in grade 1, grade 2, ER-positive and ER-negative subgroups. Survival analysis in ER-positive and ER-negative subgroups showed that IDC-L predicted a worse prognosis than IDC. In conclusion, IDC-L is a distinct histological subtype compared with IDC and ILC. Lower grade and higher ER-positive proportions mainly contribute to its better prognosis. In both ER-positive and ER-negative subgroups, IDC-L predicts worse prognosis than IDC, which suggested the inadequacy of IDC-based therapy and the need of escalated therapy.

## Introduction

Breast cancer is a heterogeneous entity with over 20 histological types^1–3^. Of these distinct histological types, invasive ductal carcinoma (IDC) is the most common one, accounting for approximately 80% of all breast cancer cases, while invasive lobular carcinoma (ILC) accounts for another 5% to 15%^4–6^. ILC differs from IDC in many respects; for example, ILC is more multifocal and bilateral and is associated with a larger tumor size, lower histological grade, higher expression of estrogen receptor (ER) and progesterone receptor (PR), lower expression of human epidermal growth factor receptor 2 (HER2) and the loss of E-cadherin expression^7–14^. These differences suggest that the development and progression of ILC are different from those of IDC^12–16^.

With the development of improved pathologic analysis techniques, IDC-L, the mixed type of IDC and ILC, has attracted increased attention. The 2012 edition of the WHO classification of breast tumors defined IDC-L as “having an ILC pattern in at least 50% of the tumor and an IDC pattern in between 10% and 49%”^17, 18^. Some previous studies have indicated that IDC-L accounts for 3–5% of all breast cancers^6, 13^. Although the features of IDC and ILC have been well-characterized, a deep understanding of IDC-L is still lacking. Some studies of IDC-L have shown that it has similar demographic and clinical characteristics to ILC^19–23^, while recent genomic analyses have indicated that IDC-L may be divided into two groups: “ILC-like” and “IDC-like”^24^. The prognostic data of IDC-L are also conflicting. One study that included 261 IDC-L patients demonstrated a similar survival outcome between patients with IDC-L and those with ILC^20^, while another study that included 140 IDC-L patients indicated that the survival outcome of IDC-L patients was similar to that of IDC patients but was significantly worse than that of ILC patients^19^. Previous studies of IDC-L are limited and contradictory because of small sample sizes, inadequate follow-up periods, a lack of adjustment for confounding factors and a lack of subgroup analyses^19–22, 25–27^. These limitations may lead to misunderstandings and inappropriate therapies for IDC-L. Therefore, it is important to clarify the clinicopathological features and prognostic factors of IDC-L within a large population, which may help doctors determine a more precise therapeutic strategy for IDC-L patients.

By using the Surveillance, Epidemiology and End Results (SEER) database, our study aimed to investigate the clinicopathological characteristics and prognosis of IDC-L by comparing it with IDC and ILC. We also conducted a subgroup analysis to compare the survival outcome of IDC-L patients with IDC and ILC in each subgroup.

## Results

### Demographic and clinical characteristics of IDC-L patients

We summarized the demographic and clinical characteristics of all 209,109 selected patients in Table 1. Compared with IDC, IDC-L patients had strikingly lower grade tumors (Grade 3 & UD: 23.4% vs 41.5%, P < 0.001, R = 0.110) and higher proportion of cases with a positive ER and PR status (93.0% vs 73.9%, P < 0.001, R = 0.133; 80.9% vs 64.0%, P < 0.001, R = 0.106, respectively). Additionally, compared with ILC, IDC-L patients were significantly younger (>50: 69.8% vs 76.3%, P < 0.001, R = 0.073) and had smaller tumor size (>5 cm: 8.1% vs 13.3%, P < 0.001, R = 0.098), fewer positive lymph nodes (≥10: 8.1% vs 10.4%, P < 0.001, R = 0.053), higher rates of lumpectomy (53% vs 46.4%, P < 0.001, R = 0.069) and similar rates of ER and PR positivity (93.0% vs 95.0%, P < 0.001, R = 0.043; 80.9% vs 79.7%, P = 0.005, R = 0.015, respectively). These data suggested that IDC-L has distinct baseline characteristics from both IDC and ILC.

**Table 1:** Baseline Characteristics of Patients with IDC, ILC or IDC-L.

Variable	IDC-L		IDC				ILC			
	n	%	n	%	P (vs IDC-L)	R ^a^	n	%	P (vs IDC-L)	R
**Year of diagnosis**					<0.001*	0.009			0.519	0.003
1998–2002	8021	41.70%	69387	40.30%			7360	42.00%		
2003–2007	11206	58.30%	102992	59.70%			10143	58.00%		
**Age at diagnosis**					<0.001*	0.027			<0.001*	0.073*
≤50 y	5806	30.20%	59488	34.50%			4149	23.70%		
>50 y	13421	69.80%	112891	65.50%			13354	76.30%		
**Race**					<0.001*	0.045			<0.001*	0.044
Black	1190	6.20%	17277	10.00%			1135	6.50%		
Others^b^	1330	6.90%	14984	8.70%			851	4.90%		
White	16618	86.40%	139487	80.90%			15456	88.30%		
Unknown	89	0.50%	631	0.40%			61	0.30%		
**Marital status**					<0.001*	0.009			0.097	0.009
Married	12102	62.90%	105956	61.50%			10821	61.80%		
Unmarried	6588	34.30%	61351	35.60%			6111	34.90%		
Unknown	537	2.80%	5072	2.90%			571	3.30%		
**Grade**					<0.001*	0.110*			<0.001*	0.134*
1	3595	18.70%	29127	16.90%			3610	20.60%		
2	10052	52.30%	67174	39.00%			7397	42.30%		
3 and UD^d^	4499	23.40%	71584	41.50%			1938	11.10%		
Unknown	1081	5.60%	4494	2.60%			4558	26.00%		
**Tumor size (cm)**					<0.001*	0.023			<0.001*	0.098*
<2	11615	60.40%	109607	63.60%			9188	52.50%		
2–5	5979	31.10%	50904	29.50%			5898	33.70%		
>5	1559	8.10%	11425	6.60%			2332	13.30%		
Unknown	74	0.40%	443	0.30%			85	0.50%		
**Positive nodes**					<0.001*	0.029			<0.001*	0.053*
0	10878	56.60%	105112	61.00%			10059	57.50%		
1–3	5002	26.00%	40558	23.50%			3945	22.50%		
4–9	1786	9.30%	13340	7.70%			1653	9.40%		
≥10	1555	8.10%	13264	7.70%			1828	10.40%		
Unknown	6	0.00%	105	0.10%			18	0.10%		
**ER status**					<0.001*	0.133*			<0.001*	0.043
Negative	1349	7.00%	44963	26.10%			867	5.00%		
Positive	17878	93.00%	127416	73.90%			16636	95.00%		
**PR status**					<0.001*	0.106*			0.005	0.015
Negative	3679	19.10%	61995	36.00%			3554	20.30%		
Positive	15548	80.90%	110384	64.00%			13949	79.70%		
**Radiation**					<0.001*	0.016			<0.001*	0.021
No	8183	42.60%	69451	40.30%			7748	44.30%		
Yes	10440	54.30%	97995	56.80%			9292	53.10%		
Unknown	604	3.10%	4933	2.90%			463	2.60%		
**Surgery type**					<0.001*	0.043			<0.001*	0.069*
No	137	0.70%	2083	1.20%			208	1.20%		
Lumpectomy	10195	53.00%	102397	59.40%			8117	46.40%		
Mastectomy	8881	46.20%	67779	39.30%			9163	52.40%		
Unknown	14	0.10%	120	0.10%			15	0.10%		

Note:

^a^Representing coefficient of contingency.

^b^Including American Indian/Alaskan native, Asian/Pacific Islander, and others-unspecified.

^c^Including divorced, separated, single (never married), and widowed.

^d^Including grade 3 and undifferentiated.

^*^Represents statistical significance. Both P < 0.001 and R > 0.05 were considered statistically significant in the comparison of the baseline characteristics. Unknown data were not included in the comparison.

Abbreviations: IDC-L: invasive ductal carcinoma with lobular features; IDC: invasive ductal carcinoma; ILC: invasive lobular carcinoma; ER: estrogen receptor; PR: progesterone receptor.

### Comparison of survival outcomes among patients with IDC-L, IDC and ILC

**Figure 1:**
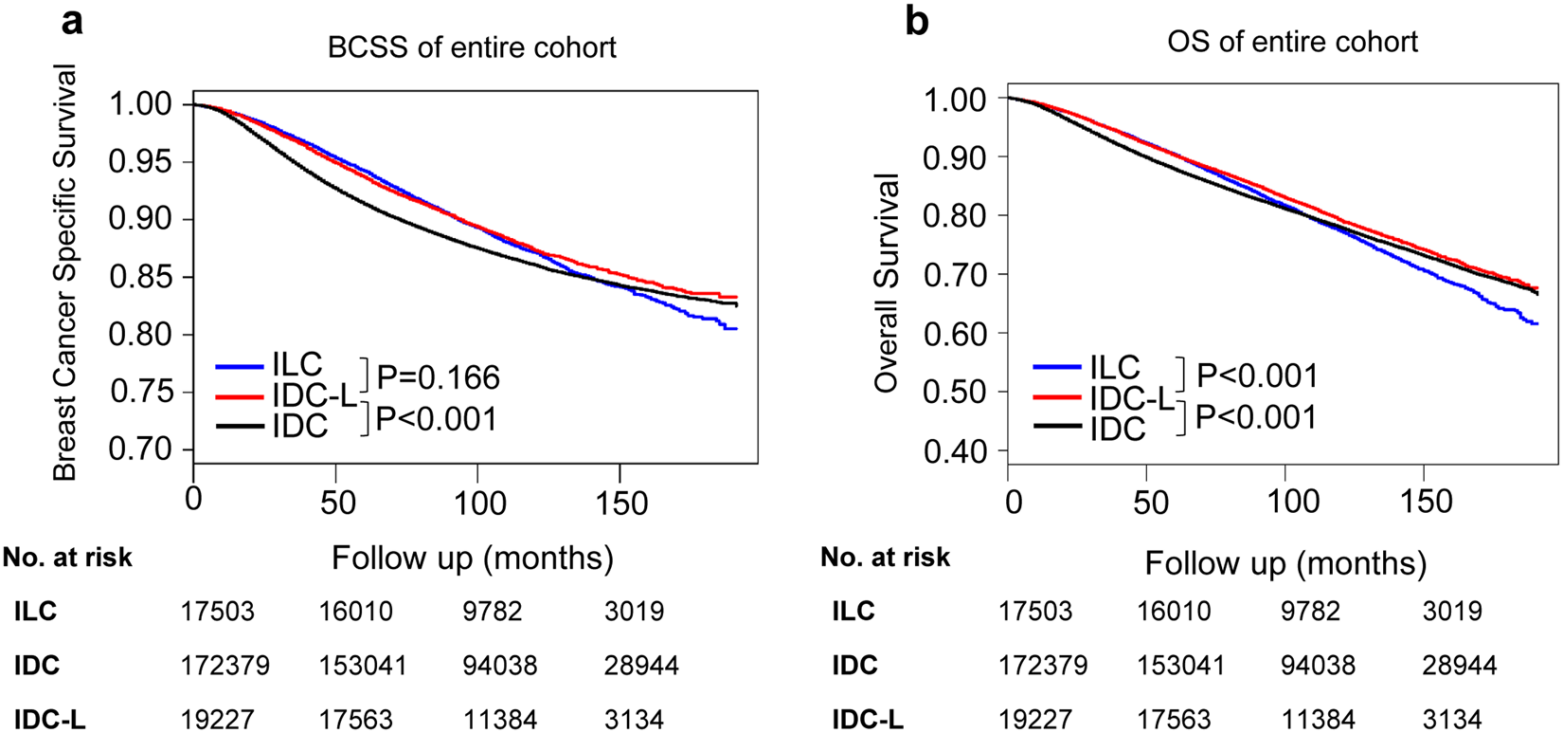
Kaplan-Meier curves of breast cancer-specific survival (BCSS) (**a**) and of overall survival (OS) (**b**) according to histological type in all patients. Log-rank tests were compared between IDC-L and IDC or ILC. Abbreviations: IDC-L: invasive ductal carcinoma with lobular features; IDC: invasive ductal carcinoma; ILC: invasive lobular carcinoma; BCSS: breast cancer-specific survival; OS: overall survival.

We compared the prognosis of these three histological types within the 11-year median follow-up period. Kaplan-Meier plots were used to evaluate the BCSS and overall survival (OS) for IDC-L, IDC and ILC (Fig. 1). In comparison with IDC patients, IDC-L patients had a significantly better BCSS and OS (both log- rank test P < 0.001). Moreover, it was obvious that the HR of IDC-L versus that of IDC increased over time according to the Scaled Schoenfeld residuals plots of the BCSS (Supplementary Fig. 1a). Additionally, compared with ILC patients, IDC-L patients exhibited a similar BCSS (log-rank test P = 0.166) and a significantly better OS (log-rank test P < 0.001). Scaled Schoenfeld residuals plots of the BCSS indicated that the HR of IDC-L versus that of ILC decreased over time (Supplementary Fig. 1b).

The Cox proportional hazards model was used to further investigate the effect of the baseline characteristics of the disease on BCSS and OS (Table 2 and Supplementary Table 1). According to the univariate analysis, several factors were significantly associated with the BCSS and OS (Supplementary Table 1). It was suggested that patients with IDC-L exhibited a better BCSS than patients with IDC (hazard ratio (HR) = 0.88, 95% confidence interval (CI): 0.83–0.92, P < 0.001), while no significant difference was observed in the BCSS between patients with IDC-L and those with ILC (HR = 0.96, 95% CI: 0.90–1.03, P = 0.262). These significant variables were then included in the multivariate analysis to confirm their prognostic effect. Most of the variables remained significant prognostic predictors in the multivariate analysis. However, after adjusting for other prognostic predictors, IDC-L was no longer an independent prognostic predictor of BCSS compared with IDC (HR = 1.00, 95% CI: 0.96–1.05, P = 0.837) or ILC (HR = 0.99, 95% CI: 0.93–1.06, P = 0.814). We concluded that although IDC-L predicted a better prognosis than IDC and ILC, it was not an independent predictor.

**Table 2:** Multivariate Analysis of Breast Cancer-specific Survival (BCSS) and Overall Survival (OS) Predictors Using a Cox Proportional Hazards Model.

Variables	BCSS		OS	
	HR (95% CI)	P	HR (95% CI)	P
**Histological type**				
IDC-L versus IDC	1.00 (0.96 to 1.05)	0.837	0.93 (0.90 to 0.97)	<0.001
IDC-L versus ILC	0.99 (0.93 to 1.06)	0.814	1.00 (0.95 to 1.05)	0.970
**Year of diagnosis**				
1998–2002	1.24 (1.20 to 1.27)	<0.001	1.22 (1.20 to 1.25)	<0.001
2003–2007	Reference			
**Age at diagnosis**				
≤50 y	0.89 (0.87 to 0.91)	<0.001	0.56 (0.55 to 0.58)	<0.001
>50 y	Reference			
**Race**				
Black	1.30 (1.26 to 1.35)	<0.001	1.28 (1.24 to 1.32)	<0.001
Others^a^	0.87 (0.83 to 0.92)	<0.001	0.81 (0.78 to 0.84)	<0.001
White	Reference			
**Marital status**				
Married	0.82 (0.80 to 0.84)	<0.001	0.69 (0.67 to 0.70)	<0.001
Unmarried^b^	Reference			
**Grade**				
1	0.50 (0.47 to 0.53)	<0.001	0. 28 (0.80 to 0.85)	<0.001
2	Reference			
3 and UD^c^	1.47 (1.43 to 1.52)	<0.001	1.23 (1.20 to 1.26)	<0.001
**Tumor size (cm)**				
<2	0.49 (0.48 to 0.51)	<0.001	0.64 (0.62 to 0.65)	<0.001
2–5	Reference			
>5	1.55 (1.49 to 1.60)	<0.001	1.49 (1.45 to 1.54)	<0.001
**Positive nodes**				
0	0.29 (0.28 to 0.30)	<0.001	0.42 (0.41 to 0.44)	<0.001
1–3	0.57 (0.54 to 0.59)	<0.001	0.60 (0.59 to 0.62)	<0.001
4–9	Reference			
≥10	1.34 (1.29 to 1.40)	<0.001	1.40 (1.35 to 1.45)	<0.001
**ER status**				
Negative	1.36 (1.31 to 1.41)	<0.001	1.19 (1.15 to 1.22)	<0.001
Positive	Reference			
**PR status**				
Negative	1.37 (1.32 to 1.42)	<0.001	1.23 (1.20 to 1.27)	<0.001
Positive	Reference			
**Radiation**				
No	1.16 (1.13 to 1.20)	<0.001	1.29 (1.26 to 1.32)	<0.001
Yes	Reference			
**Surgery type**				
No	1.42 (1.32 to 1.54)	<0.001	1.34 (1.25 to 1.44)	<0.001
Lumpectomy	0.85 (0.82 to 0.87)	<0.001	0.87 (0.85 to 0.89)	<0.001
Mastectomy	Reference			

Note:

^a^Including American Indian/Alaskan native, Asian/Pacific Islander, and others-unspecified.

^b^Including divorced, separated, single (never married), and widowed.

^c^Including grade 3 and undifferentiated.

Abbreviations: BCSS: breast cancer-specific survival; OS: overall survival; HR: hazard ratio; CI: confidence interval; IDC-L: invasive ductal carcinoma with lobular features; IDC: invasive ductal carcinoma; ILC: invasive lobular carcinoma; ER: estrogen receptor; PR: progesterone receptor.

### Subgroup analysis

**Figure 2:**
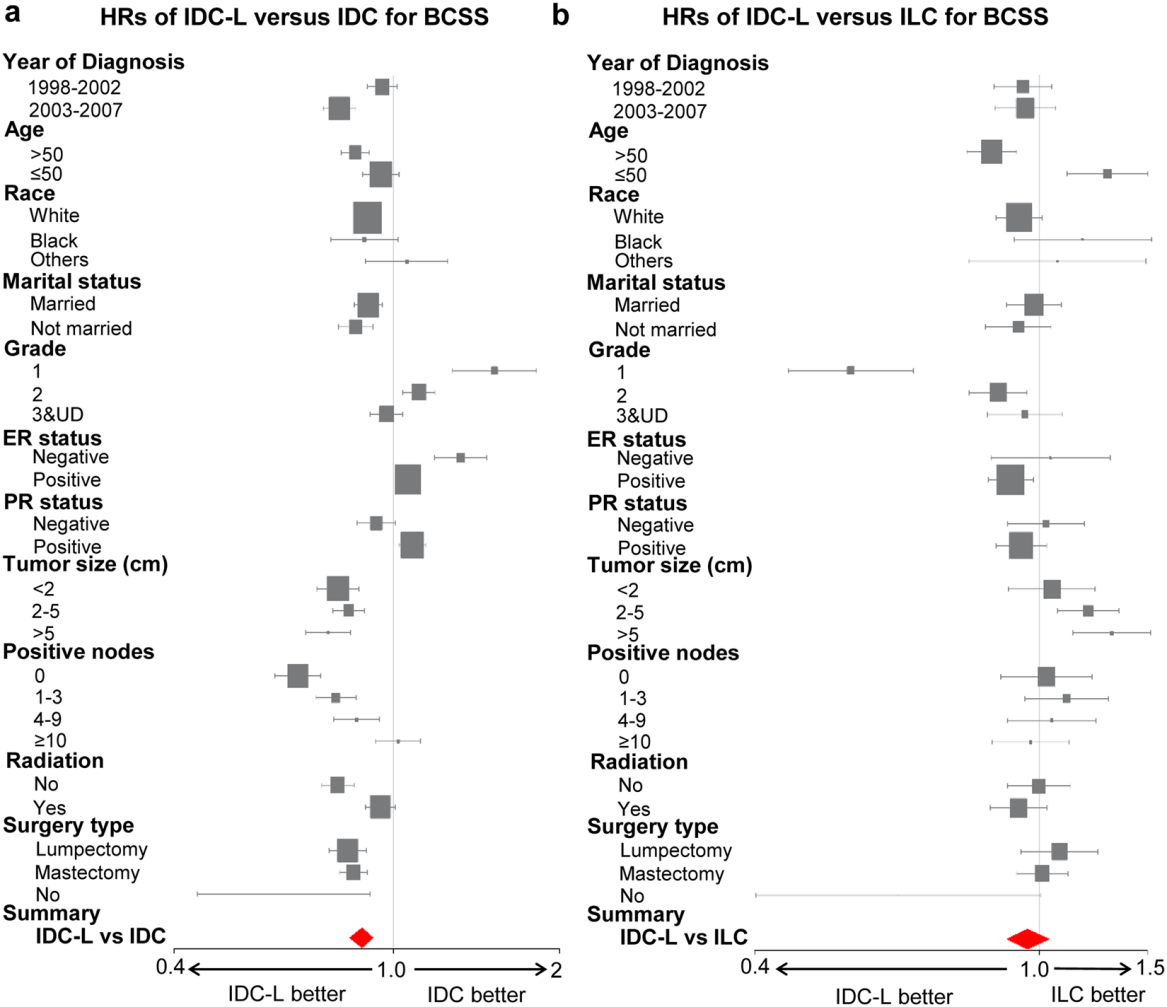
Forest plots of hazard ratios (HRs) of IDC-L versus IDC for BCSS (**a**) and of IDC-L versus ILC for BCSS (**b**) according to the subgroup analysis. The X-axis shows the HRs and 95% confidence intervals (CIs) of each subgroup. The size of the boxes represents the relative number of patients in each subgroup. Abbreviations: HRs: hazard ratios; BCSS: breast cancer-specific survival; OS: overall survival; IDC-L: invasive ductal carcinoma with lobular features; IDC: invasive ductal carcinoma; ILC: invasive lobular carcinoma; ER: estrogen receptor; PR: progesterone receptor.

In order to investigate whether IDC-L predicted homogeneous prognosis when stratified by different clinical parameters, we conducted a subgroup analysis to compare the BCSS among IDC-L, IDC and ILC in each subgroup. Forest plots of HRs in the univariate Cox analysis summarized the exploratory subgroup analysis of the BCSS and are shown in Fig. 2. Compared with IDC, IDC-L no longer had lower HRs for BCSS in some subgroups. IDC-L had higher HRs for BCSS than IDC in the grade 1 (HR = 1.52, 95% CI: 1.28–1.82, P < 0.001) and grade 2 (HR = 1.11, 95% CI: 1.04–1.19, P = 0.002) subgroups. IDC-L was also found to be a risk factor for a low BCSS rate compared with IDC in both the ER-positive (HR = 1.06, 95% CI: 1.01–1.11, P = 0.02) and ER-negative (HR = 1.32, 95% CI: 1.19–1.47, P < 0.001) subgroups. In addition, consistent with the analysis of the entire study population, the HRs of IDC-L versus those of ILC for BCSS were not significant in most subgroups. However, IDC-L was found to predict a better BCSS than ILC in patients older than 50 years of age (HR = 0.86, 95% CI: 0.79–0.93, P < 0.001) and in those with grade 1 (HR = 0.55, 95% CI: 0.45–0.67, P < 0.001) and grade 2 tumors (HR = 0.88, 95% CI: 0.80–0.96, P = 0.005), while this effect was reversed in patients younger than 50 years of age (HR = 1.25, 95% CI: 1.09–1.41, P < 0.001) and in those with tumor sizes between 2–5 cm (HR = 1.17, 95% CI: 1.06–1.29, P = 0.002). These data suggested that some clinicopathological markers, such as tumor grade and ER status, were important confounders in determining the prognosis of IDC-L patients.

### Survival analysis in the ER-positive and ER-negative subgroups

Although Kaplan-Meier plots and the univariate Cox analysis indicated that IDC-L was associated with a better BCSS in entire cohort, a subgroup analysis suggested that the BCSS was not the same in some specific subgroups. As ER is an important therapeutic target in clinical practice, we conducted an additional survival analysis of the ER-positive and ER-negative subgroups.

**Figure 3:**
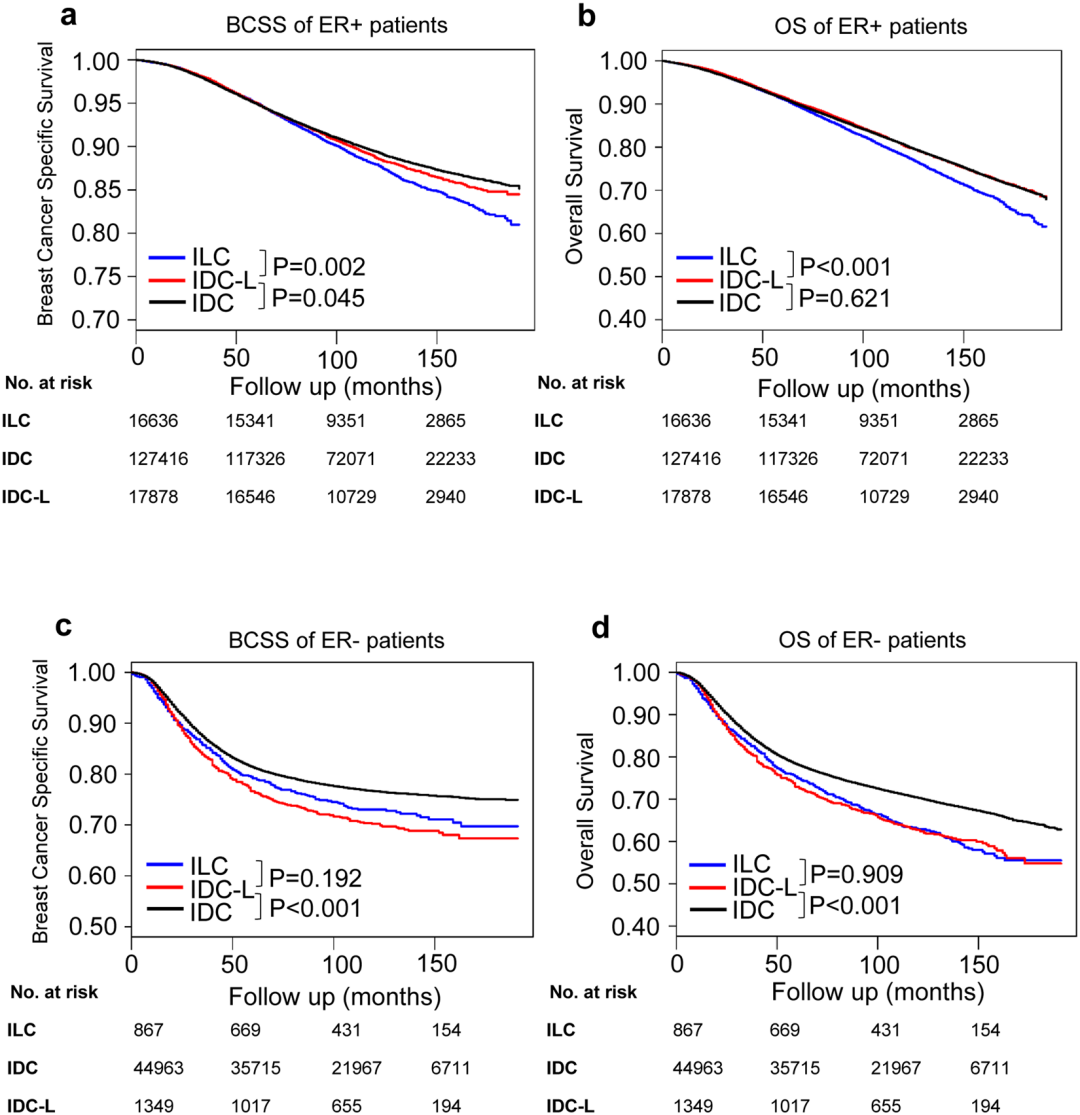
Kaplan-Meier curves of breast cancer-specific survival (BCSS) (left) and of overall survival (OS) (right) according to histological type in the ER-positive subgroup (**a**,**b**) and ER-negative subgroup (**c**,**d**). Log-rank tests were compared between IDC-L and IDC or ILC. Abbreviations: IDC-L: invasive ductal carcinoma with lobular features; IDC: invasive ductal carcinoma; ILC: invasive lobular carcinoma; BCSS: breast cancer-special survival; OS: overall survival.

Kaplan-Meier plots showed that IDC-L patients in the ER-positive subgroup had a moderate BCSS, which was significantly worse than that of IDC patients (log-rank test P = 0.045) and better than that of ILC patients (log-rank test P = 0.002) (Fig. 3a). The difference in survival among these patients in terms of BCSS disappeared after the adjustment for other clinicopathological data (Supplementary Table 2). Additionally, the OS analysis of patients in the ER-positive subgroup demonstrated that the OS of IDC-L patients was similar to that of IDC patients (log-rank test P = 0.621) and was significantly better than that of ILC patients (log-rank test P < 0.001) (Fig. 3b). Moreover, IDC-L patients in the ER-negative subgroup had a significantly worse BCSS and OS than IDC patients (both log-rank test P < 0.001), while they had a similar BCSS and OS compared with ILC patients (log-rank test P = 0.192, log-rank test P = 0.909, respectively) (Fig. 3c,d). The survival disadvantage of IDC-L still existed even after adjusting for other clinicopathological data (Supplementary Table 3). These results demonstrated distinct prognostic features between the entire study population and the ER-positive and ER-negative subgroups. In the ER-negative subgroup, IDC-L independently predicted worse prognosis than IDC while in the ER-positive subgroup, further research were needed to investigate whether there exists a smaller subgroup of IDC-L patients that independently predicted worse prognosis than IDC patients.

## Discussion

In this study, we retrospectively investigated the clinicopathological characteristics and prognostic features of IDC-L through a comparison of IDC-L with IDC and ILC. The results suggested that IDC-L has baseline characteristics that are distinct from those of IDC and ILC. A survival analysis indicated that IDC-L was associated with a significantly better BCSS than IDC and tended to have a better BCSS than ILC. However, the difference in BCSS between IDC-L and IDC disappeared after adjusting for confounding factors. The subgroup analysis that followed revealed that a different distribution of tumor grade and ER status accounted for the better survival of IDC-L patients than that of IDC patients. Furthermore, we found that when stratified according to their ER status, patients with IDC-L had a worse BCSS than those with IDC, which was observed for patients in both the ER-positive and ER-negative subgroups.

As the largest analysis of IDC-L to date, our research took advantage of the high number of SEER datasets to further investigate the clinicopathological characteristics of IDC-L. The proportions of the three histological types in our study were 82.4% (IDC), 8.4% (ILC) and 9.2% (IDC-L). Although the proportion of IDC-L was slightly higher than in some other studies^19–22^, we confirmed the accuracy of the data in the selection of patients with each subtype in our study through other SEER datasets based on the research of Li *et al*.^5^. Through a comparison of the clinicopathological characteristics among the three histological types, we observed that IDC-L not only shared similarities but also shared differences with IDC and ILC. It is therefore more reasonable to consider that the clinicopathological characteristics of IDC-L are intermediate and between those of IDC and ILC.

According to the survival analysis of the entire study population, our study demonstrated that IDC-L patients exhibited a significantly better BCSS than IDC and tended to exhibit a better BCSS than ILC patients. We attributed the differences between our study and several others^19, 21, 22^ to the small sample sizes, short follow-up periods and different definitions of IDC-L in those studies. Furthermore, we analyzed the change in the HRs over time for IDC-L versus IDC and for IDC-L versus ILC. Scaled Schoenfeld residuals plots, which indicated the increased HR of IDC-L versus IDC over time, reminded us to be cautious with the long-term risk of IDC-L despite its better prognosis. In addition, the significantly decreased HR of IDC-L versus ILC over time suggested that the long-term prognosis of IDC-L was significantly better than that of ILC.

Our study also focused on deeper subgroup analysis of IDC, ILC and IDC-L. A multivariate Cox analysis indicated that IDC-L was not an independent predictor of a better BCSS compared with IDC. The subgroup analysis that followed focused on the identification of the underlying factors that contribute to this phenomenon and revealed that tumor grade and ER status were important confounders. IDC-L patients were more likely to have lower grade tumors and ER-positive disease, and the survival advantage of low tumor grade and ER positivity themselves contributed to the better BCSS of IDC-L versus that of IDC. Additionally, the forest plot showed that compared with IDC, IDC-L no longer predicted lower HRs in the ER-positive and ER-negative subgroups. These findings indicated that it was too ambiguous to consider IDC-L as a histological type with a better prognosis than IDC, and thus offer these patients with similar or de-escalated therapies compared with IDC patients. In contrast, more individualized therapy for IDC-L should be considered based on the personal characteristics of each patient, such as ER status. Kaplan-Meier plots confirmed that the BCSS of IDC-L was worse than that of IDC for patients in both the ER-positive and ER-negative subgroups. The survival disadvantage of IDC-L patients in the ER-negative subgroup was still present even after we adjusted for other clinicopathological data. Current treatment for IDC-L is based on evidence from studies of IDC^28^, but our analysis suggests that this treatment might be inadequate for IDC-L patients in both the ER-positive and ER-negative subgroup. For ER-negative IDC-L patients, escalated chemotherapy may be considered after comprehensive evaluation. For ER-positive IDC-L patients, we need further research to investigate whether there exists a smaller subgroup that may need extended endocrine therapy, chemotherapy or ovarian function inhibition, even evaluated as at low risk in IDC-based guidelines.

Our research also has several limitations. First, it is a retrospective study and may have some potential selection bias. In addition, previous studies^19–22^ suggested that IDC-L and ILC were associated with a significantly lower percentage of HER2-positive cases compared with IDC, which might have affected the outcome, but HER2 information in the SEER datasets was not available until 2010. To evaluate a long follow-up period, we included patients who were diagnosed between 1998 and 2007, and therefore, we lacked information on their HER2 status. Furthermore, as the understanding of IDC-L in our study focused on the clinicopathological characteristics, further studies are needed to investigate its molecular features and to reveal the genetic features and biological nature of IDC-L.

In conclusion, IDC-L is a histological subtype that is distinct from IDC and ILC with respect to clinicopathological characteristics and prognostic features. Although IDC-L is associated with a survival advantage in the entire study population, it predicts a worse prognosis than IDC for patients both in the ER-positive and ER-negative subgroup. The high proportion of ER-positive status of IDC-L patients and the survival advantage of ER-positive status itself contribute to this phenomenon. Therefore, an escalated therapeutic strategy may be considered for both ER-positive and ER-negative IDC-L patients.

## Methods

### Database

Data for this study were obtained from the recent SEER18 registry research database (November 2015 Submission). The SEER18 database contains data from the SEER13 registries (Atlanta, Connecticut, Detroit, Hawaii, Iowa, New Mexico, San Francisco-Oakland, Seattle-Puget Sound, Utah, Los Angeles, San Jose-Monterey, rural Georgia, and the Alaska Native Tumor Registry) and the registries of greater California, Kentucky, Louisiana, New Jersey, and greater Georgia. The SEER database of the National Cancer Institute (NCI) is the largest population-based cancer registry in the United States and covers approximately 28% of the population (http://seer.cancer.gov/about/).

### Study population

Our data were obtained from the SEER database released in April 2016, which includes data from 18 population-based registries (1973–2013). The inclusion criteria were as follows: female patients, diagnosis year from 1998 to 2007, histological grades I-IV (Grade IV is the undifferentiated (UD) type), American Joint Committee on Cancer (AJCC) stages I-III, pathologic confirmation of infiltrating ductal carcinoma-not otherwise specified (IDC-NOS, ICD-O-3 8500/3), lobular carcinoma-not otherwise specified (ILC-NOS, ICD-O-3 8520/3), and infiltrating duct and lobular carcinoma (IDC-L, ICD-O-3 8522/3), unilateral breast cancer, breast cancer as the first and only cancer diagnosis, diagnosis not obtained from a death certificate or autopsy, only one primary site, and known ER and PR status. In all, 209109 patients were included, including 172379 IDC patients (82.4%), 17503 ILC patients (8.4%) and 19227 IDC-L patients (9.2%).

An analysis of the demographic and clinical characteristics of the IDC, ILC and IDC-L subtypes included the year of diagnosis, age at diagnosis, race, marital status, grade, ER and PR status, tumor size, number of positive nodes, radiation type and surgery type. We considered the year of diagnosis as a binary variable and classified it into two groups: 1998–2002 and 2003–2007. The age at diagnosis was also considered a binary variable: <50 and ≥50. Marital status was summarized, and patients were classified as married, not married or unknown. Grade 3 and undifferentiated grade were merged into a single group. Moreover, tumor size was classified into 4 groups: <2 cm, 2–5 cm, >5 cm or unknown, while the number of positive lymph nodes was categorized into 5 groups: 0, 1–3, 4–9, ≥10 or unknown. In addition, the types of radiation were summarized as yes, no or unknown; the types of surgery were classified as no surgery, lumpectomy, mastectomy or unknown. Detailed classification information is described in Table 1. All unknown data were excluded from the Cox analysis and subgroup analysis but were included in the generation of the Kaplan-Meier curves.

### Statistical analysis

The demographic and clinical characteristics of the included cases were compared across groups by the Pearson Chi-square test or Fisher’s exact test for categorical nominal data and by the Cochran-Mantel Haenszel (CMH) Chi-square test for categorical ordinal data. As a large patient sample size might lead to failure of the Chi-square test, we added a contingency coefficient (R) as a reference. The BCSS and OS were considered as the primary and secondary outcomes of our study, respectively. BCSS was defined as the time from the date of diagnosis to the date of death caused by breast cancer. The OS was defined as the time from the date of diagnosis to the date of death from any cause. Kaplan-Meier curves and log-rank tests were generated with the function “Surv” and “survfit” (R package: survival and rms). Scaled Schoenfeld residuals plots were drawn with the function “cox.zph” (R package: survival). Additionally, univariate and multivariate Cox proportional hazard models with HRs and 95% CIs were applied to estimate the factors associated with the BCSS and OS. Subgroup analysis and forest plots were generated with the function “forestplot” (R package: forestplot). All statistical analyses were performed using R version 3.2.2 (R Foundation for Statistical Computing, Vienna, Austria) and SPSS version 17.0 (IBM SPSS Statistics, Chicago, IL, USA). A two-sided P value < 0.001 and contingency coefficient (R) > 0.05 were considered statistically significant in the comparison of clinicopathological characteristics. Two-sided P values < 0.05 were considered statistically significant in other tests.

## Electronic supplementary material

Supplementary Material
