# Supplementary material for: Mixed invasive ductal and lobular carcinoma has distinct clinical features and predicts worse prognosis when stratified by estrogen receptor status
Source: Sci Rep. 2017 Sep 4;7:10380. doi: 10.1038/s41598-017-10789-x (PMC5583173; doi:10.1038/s41598-017-10789-x)
Supplement: Supplementary file 1 — Supplementary Material [file 41598_2017_10789_MOESM1_ESM.pdf]

Mixed invasive ductal and lobular carcinoma has distinct clinical features and predicts worse prognosis when stratified by estrogen receptor status

Yi Xiao MD[1,2], Ding Ma MD [1,2], Miao Ruan MD [2,3], Shen Zhao MD [1,2], Xi-Yu Liu MD [1,2], Yi-Zhou Jiang MD [1,2] and Zhi-Ming Shao MD, PhD [1,2,4]

Authors' Affiliations and Addresses:

1 Department of Breast Surgery, Fudan University Shanghai Cancer Center; Cancer Institute, Fudan University Shanghai Cancer Center, 399 Ling-Ling Road, Shanghai 200032, People's Republic of China

2 Department of Oncology, Shanghai Medical College, Fudan University, P.R. China

3 Department of Pathology, Fudan University Shanghai Cancer Center, P.R. China.

4 Institutes of Biomedical Sciences, Fudan University, Shanghai, P.R. China

Yi Xiao, Ding Ma and Miao Ruan contributed equally to this work.

***Correspondence to:**

Zhi-Ming Shao, MD, PhD or Yi-Zhou Jiang, MD

Department of Breast Surgery, Fudan University Shanghai Cancer Center

Cancer Institute, Fudan University Shanghai Cancer Center;

Department of Oncology, Shanghai Medical College, Fudan University

270 Dong-An Road, Shanghai, 200032, P.R. China

Tel: +86-21-64175590

Fax: +86-21-64434556

Email: zhiminqshao@gmail.com or yizhoujiang@fudan.edu.cn

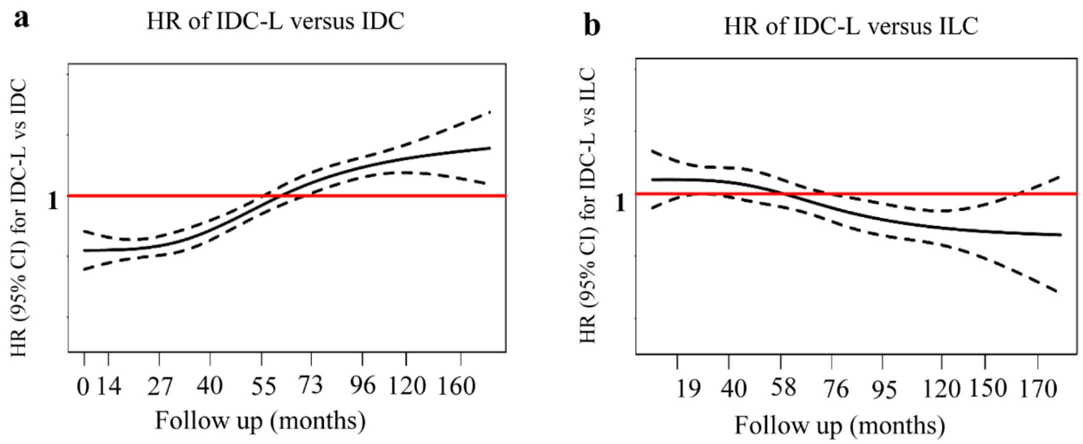

Supplementary Fig. 1. Scaled Schoenfeld residuals plots to estimate the change of HR for breast cancer specific death over time for IDC-L versus IDC (a) and IDC-L versus ILC (b). The solid line represents point estimate of HR; the dotted line represents the 95% confidence interval of HR. Abbreviations: HR: hazard ratio; CI: confidence interval; IDC-L: invasive ductal carcinoma with lobular features; IDC: invasive ductal carcinoma; ILC: invasive lobular carcinoma.

Supplementary Table 1. Univariate Analysis of Breast Cancer Specific Survival (BCSS) and Overall Survival (OS) Predictors Using Cox Proportional Hazards Model

Variables	BCSS		OS	
	HR (95% CI)	P	HR (95% CI)	P
**Histological type**				
**IDC-L versus IDC**	1.00 (0.96 to 1.05)	0.837	0.93 (0.90 to 0.97)	<0.001
**IDC-L versus ILC**	0.99 (0.93 to 1.06)	0.814	1.00 (0.95 to 1.05)	0.970
**Year of diagnosis**				
**1998-2002**	1.24 (1.20 to 1.27)	<0.001	1.22 (1.20 to 1.25)	<0.001
**2003-2007**	Reference			
**Age at diagnosis (years)**				
**≤50**	0.89 (0.87 to 0.91)	<0.001	0.56 (0.55 to 0.58)	<0.001
**>50**	Reference			
**Race**				
**Black**	1.30 (1.26 to 1.35)	<0.001	1.28 (1.24 to 1.32)	<0.001
**Others** [a]	0.87 (0.83 to 0.92)	<0.001	0.81 (0.78 to 0.84)	<0.001
**White**	Reference			
**Marital status**				
**Married**	0.82 (0.80 to 0.84)	<0.001	0.69 (0.67 to 0.70)	<0.001
**Unmarried** [b]	Reference			
**Grade**				
**1**	0.50 (0.47 to 0.53)	<0.001	0. 28 (0.80 to 0.85)	<0.001
**2**	Reference			
**3 and UD** [c]	1.47 (1.43 to 1.52)	<0.001	1.23 (1.20 to 1.26)	<0.001
**Tumor size (cm)**				
**<2**	0.49 (0.48 to 0.51)	<0.001	0.64 (0.62 to 0.65)	<0.001
**2-5**	Reference			
**>5**	1.55 (1.49 to 1.60)	<0.001	1.49 (1.45 to 1.54)	<0.001
**Positive nodes**				
**0**	0.29 (0.28 to 0.30)	<0.001	0.42 (0.41 to 0.44)	<0.001
**1-3**	0.57 (0.54 to 0.59)	<0.001	0.60 (0.59 to 0.62)	<0.001
**4-9**	Reference			
**≥10**	1.34 (1.29 to 1.40)	<0.001	1.40 (1.35 to 1.45)	<0.001
**ER status**				
**Negative**	1.36 (1.31 to 1.41)	<0.001	1.19 (1.15 to 1.22)	<0.001
**Positive**	Reference			
**PR status**				
**Negative**	1.37 (1.32 to 1.42)	<0.001	1.23 (1.20 to 1.27)	<0.001
**Positive**	Reference			
**Radiation**				
**No**	1.16 (1.13 to 1.20)	<0.001	1.29 (1.26 to 1.32)	<0.001
**Yes**	Reference			
**Surgery type**				
**No**	1.42 (1.32 to 1.54)	<0.001	1.34 (1.25 to 1.44)	<0.001
**Lumpectomy**	0.85 (0.82 to 0.87)	<0.001	0.87 (0.85 to 0.89)	<0.001
**Mastectomy**	Reference			

Note:

a Including American Indian/Alaskan native, and Asian/Pacific Islander, and others-unspecified.

b Including divorced, separated, single (never married), and widowed.

c Including grade 3 and undifferentiated.

Abbreviations: BCSS: breast cancer specific survival; OS: overall survival; HR: hazard ratio; CI: confidence interval; IDC-L: invasive ductal carcinoma with lobular features; IDC: invasive ductal carcinoma; ILC: invasive lobular carcinoma; ER: estrogen receptor; PR: progesterone receptor.

Supplementary Table 2. Multivariate Analysis of Breast Cancer Specific Survival (BCSS) and Overall Survival (OS) Predictors in ER-positive Patients Using Cox Proportional Hazards Model

Variables	BCSS		OS	
	HR (95% CI)	P	HR (95% CI)	P
**Histological type**				
**IDC-L versus IDC**	0.98 (0.93 to 1.03)	0.472	0.91 (0.88 to 0.95)	<0.001
**IDC-L versus ILC**	0.97 (0.91 to 1.05)	0.489	0.99 (0.93 to 1.04)	0.600
**Year of diagnosis**				
**1998-2002**	1.20 (1.15 to 1.24)	<0.001	1.19 (1.16 to 1.22)	<0.001
**2003-2007**	Reference			
**Age at diagnosis (years)**				
**≤50**	0.88 (0.85 to 0.91)	<0.001	0.50 (0.48 to 0.51)	<0.001
**>50**	Reference			
**Race**				
**Black**	1.42 (1.35 to 1.50)	<0.001	1.34 (1.29 to 1.39)	<0.001
**Others** [a]	0.91 (0.85 to 0.96)	0.002	0.82 (0.78 to 0.86)	<0.001
**White**	Reference			
**Marital status**				
**Married**	0.81 (0.78 to 0.84)	<0.001	0.65 (0.64 to 0.67)	<0.001
**Unmarried** [b]	Reference			
**Grade**				
**1**	0.51 (0.48 to 0.55)	<0.001	0. 82 (0.79 to 0.85)	<0.001
**2**	Reference			
**3 and UD** [c]	1.62 (1.56 to 1.68)	<0.001	1.30 (1.26 to 1.33)	<0.001
**Tumor size (cm)**				
**<2**	0.46 (0.44 to 0.48)	<0.001	0.64 (0.62 to 0.66)	<0.001
**2-5**	Reference			
**>5**	1.45 (1.39 to 1.52)	<0.001	1.42 (1.36 to 1.48)	<0.001
**Positive nodes**				
**0**	0.29 (0.27 to 0.30)	<0.001	0.46 (0.44 to 0.47)	<0.001
**1-3**	0.56 (0.53 to 0.59)	<0.001	0.61 (0.59 to 0.64)	<0.001
**4-9**	Reference			
**≥10**	1.42 (1.34 to 1.49)	<0.001	1.48 (1.41 to 1.54)	<0.001
**PR status**				
**Negative**	1.41 (1.35 to 1.47)	<0.001	1.24 (1.20 to 1.27)	<0.001
**Positive**	Reference			
**Radiation**				
**No**	1.17 (1.13 to 1.21)	<0.001	1.32 (1.29 to 1.36)	<0.001
**Yes**	Reference			
**Surgery type**				
**No**	1.41 (1.27 to 1.57)	<0.001	1.23 (1.13 to 1.35)	<0.001
**Lumpectomy**	0.84 (0.81 to 0.87)	<0.001	0.86 (0.84 to 0.89)	<0.001
**Mastectomy**	Reference			

Note:

a Including American Indian/Alaskan native, and Asian/Pacific Islander, and others-unspecified.

b Including divorced, separated, single (never married), and widowed.

c Including grade 3 and undifferentiated.

Abbreviations: BCSS: breast cancer specific survival; OS: overall survival; HR: hazard ratio; CI: confidence interval; IDC-L: invasive ductal carcinoma with lobular features; IDC: invasive ductal carcinoma; ILC: invasive lobular carcinoma; ER: estrogen receptor; PR: progesterone receptor.

Supplementary Table 3. Multivariate Analysis of Breast Cancer Specific Survival (BCSS) and Overall Survival (OS) Predictors in ER-negative Patients Using Cox Proportional Hazards Model

Variables	BCSS		OS	
	HR (95% CI)	P	HR (95% CI)	P
**Histological type**				
**IDC-L versus IDC**	1.13 (1.01 to 1.26)	0.030	1.11 (1.01 to 1.23)	0.031
**IDC-L versus ILC**	1.00 (0.83 to 1.22)	0.980	0.96 (0.81 to 1.13)	0.628
**Year of diagnosis**				
**1998-2002**	1.28 (1.23 to 1.33)	<0.001	1.26 (1.22 to 1.31)	<0.001
**2003-2007**	Reference			
**Age at diagnosis (years)**				
**≤50**	0.91 (0.87 to 0.95)	<0.001	0.71 (0.68 to 0.73)	<0.001
**>50**	Reference			
**Race**				
**Black**	1.20 (1.14 to 1.26)	<0.001	1.21 (1.15 to 1.26)	<0.001
**Others** [a]	0.82 (0.76 to 0.89)	<0.001	0.80 (0.74 to 0.85)	<0.001
**White**	Reference			
**Marital status**				
**Married**	0.84 (0.80 to 0.87)	<0.001	0.77 (0.74 to 0.79)	<0.001
**Unmarried** [b]	Reference			
**Grade**				
**1**	0.58 (0.47 to 0.70)	<0.001	0. 74 (0.64 to 0.85)	<0.001
**2**	Reference			
**3 and UD** [c]	1.16 (1.10 to 1.23)	<0.001	1.08 (1.03 to 1.13)	<0.001
**Tumor size (cm)**				
**<2**	0.55 (0.52 to 0.58)	<0.001	0.63 (0.60 to 0.65)	<0.001
**2-5**	Reference			
**>5**	1.66 (1.57 to 1.75)	<0.001	1.59 (1.51 to 1.67)	<0.001
**Positive nodes**				
**0**	0.30 (0.28 to 0.32)	<0.001	0.37 (0.35 to 0.39)	<0.001
**1-3**	0.59 (0.55 to 0.62)	<0.001	0.60 (0.57 to 0.64)	<0.001
**4-9**	Reference			
**≥10**	1.33 (1.24 to 1.33)	<0.001	1.24 (1.17 to 1.32)	<0.001
**PR status**				
**Negative**	1.20 (1.10 to 1.30)	<0.001	1.17 (1.09 to 1.26)	<0.001
**Positive**	Reference			
**Radiation**				
**No**	1.15 (1.10 to 1.20)	<0.001	1.24 (1.19 to 1.29)	<0.001
**Yes**	Reference			
**Surgery type**				
**No**	1.45 (1.29 to 1.63)	<0.001	1.49 (1.34 to 1.65)	<0.001
**Lumpectomy**	0.88 (0.84 to 0.92)	<0.001	0.88 (0.85 to 0.92)	<0.001
**Mastectomy**	Reference			

Note:

a Including American Indian/Alaskan native, and Asian/Pacific Islander, and others-unspecified.

b Including divorced, separated, single (never married), and widowed.

c Including grade 3 and undifferentiated.

Abbreviations: BCSS: breast cancer specific survival; OS: overall survival; HR: hazard ratio; CI: confidence interval; IDC-L: invasive ductal carcinoma with lobular features; IDC: invasive ductal carcinoma; ILC: invasive lobular carcinoma; ER: estrogen receptor; PR: progesterone receptor.
